# Network-Based Biomarkers for Cold Coagulation Blood Stasis Syndrome and the Therapeutic Effects of Shaofu Zhuyu Decoction in Rats

**DOI:** 10.1155/2013/901943

**Published:** 2013-10-21

**Authors:** Shulan Su, Jinao Duan, Wenxia Cui, Erxing Shang, Pei Liu, Gang Bai, Sheng Guo, Dawei Qian, Yuping Tang

**Affiliations:** ^1^Jiangsu Key Laboratory for High Technology Research of TCM Formulae, Nanjing University of Chinese Medicine, Nanjing 210046, China; ^2^College of Pharmacy, State Key Laboratory of Medicinal Chemical Biology, Tianjin Key Laboratory of Molecular Drug Research, Nankai University, Tianjin 300071, China

## Abstract

In this study, the reverse docking methodology was applied to predict the action targets and pathways of Shaofu Zhuyu decoction (SFZYD) bioactive ingredients. Furthermore, Traditional Chinese Medicine (TCM) cold coagulation blood stasis (CCBS) syndrome was induced in female Sprague-Dawley rats with an ice-water bath and epinephrine, and SFZYD was used to treat CCBS syndrome. A metabolomic approach was used to evaluate changes in the metabolic profiles and to analyze the pharmacological mechanism of SFZYD actions. Twenty-three potential protein targets and 15 pathways were discovered, respectively; among these, pathways are associated with inflammation and immunological stress, hormone metabolism, coagulation function, and glycometabolism. There were also changes in the levels of endogenous metabolites of LysoPCs and glucuronides. Twenty endogenous metabolites were identified. Furthermore, the relative quantities of 6 endogenous metabolites in the plasma and 5 in the urine were significantly affected by SFZYD (*P* < 0.05). The pharmacological mechanism of SFZYD was partially associated with glycerophospholipid metabolism and pentose and glucuronate interconversions. In conclusion, our findings demonstrated that TCM CCBS pattern induced by ice water and epinephrine was complex and related to multiple metabolic pathways. SFZYD did regulate the TCM CCBS by multitargets, and biomarkers and SFZYD should be used for the clinical treatment of CCBS syndrome.

## 1. Introduction

Traditional Chinese medicine (TCM) is guided by the theory of traditional Chinese medical science for clinical application. TCM can be characterized as being holistic, with an emphasis on regulation of the integrity of the human body and the interaction between individuals and their environment. Multiple natural therapeutic methods for patient's management were applied in TCM; among these, the herbal formula was the most typical treatment. Most herbal medicines are prescribed in combination to obtain synergistic effects or diminish possible adverse reactions [1]. This medical approach has played on increasingly important role in evidence-based personalized medicine, which is a new trend and a hot research topic of medical development.

Based on the theory of TCM, which is rooted in the philosophy of treating the entire body as a whole, multipathogenic factors, such as cold coagulation, Qi stagnation, and blood insufficiency, are considered to be the main causes of many diseases or syndromes. In TCM, there are several different disharmony patterns (named ZHENG) such as the syndrome of blood stasis, syndrome of Qi stagnation, and Yin/Yang deficiency syndrome [2]. The TCM ZHENG as a diagnostic approach may provide an invaluable guidance for therapeutic choices and personalized disease management, not only in traditional medical practices but also in modern healthcare systems [3].

Cold coagulation blood stasis (CCBS) is primarily induced by cold-evil, and as a subtype of blood stasis syndrome, it is a critical pattern for many diseases, especially in women. The pathologic mechanisms of CCBS syndrome were recently found to be related to the changes in hemorheological properties including high-blood viscosity, increased erythrocyte aggregation, increased blood sedimentation, decreased erythrocyte deformability, decreased hematocrit, microcirculation disturbance, and coagulant function abnormality [4]. Further research revealed that blood stasis syndrome is closely related to inflammatory factors and the immune response. Inflammatory- and immune-related genes are remarkably dominant in the gene expression profiles of blood stasis syndrome, which may explain the functions of inflammation and the immune response in the occurrence and development of this syndrome [5]. Moreover, it was reported that the occurrence of vasomotor dysfunction in the ovaries and the levels of reproductive hormone decrease in patients with blood stasis syndrome [6], but the complex pathological mechanisms and metabolite profiling changes in CCBS syndrome remain incompletely elucidated.

The famous Chinese herbal formula, Shaofu Zhuyu decoction (SFZYD), which was originally identified by the “*Correction of Errors in Medical Classics*” compiled by Qing-ren Wang in the Qing dynasty (A.D. 1830), was utilized in the clinic for approximately 200 years to treat blood stasis syndrome in gynecological diseases such as dysmenorrhea, amenorrhea, or menoxenia. SFZYD is considered an effective prescription for the treatment of CCBS syndrome, and it is composed of ten crude drugs: *Angelicae sinensis Radix*, *Chuanxiong Rhizoma*, *Cinnamomi Cortex*, *Foeniculi Fructus*, *Zingiberis Rhizoma*, Myrrha, *Trogopterpri Faeces*, *Typhae Pollen*, *Paeoniae Radix Rubra*, and *Corydalis Rhizoma* [7]. In the clinic, SFZYD was reported to have over 90% efficacy for the treatment of primary dysmenorrhea with CCBS syndrome [8, 9]. However, the therapeutic mechanisms of SFZYD are still unknown and require a comprehensive investigation.

Metabolomics, which is based on the dynamic changes of low molecular weight metabolites in organisms, indicates the overall physiological status in response to pathophysiological stimuli or genetic, environmental, or lifestyle factors [10]. Metabolites often mirror the end result of genomic and protein perturbations in a disease, and they are most closely associated with phenotypic changes. Furthermore, the pathogenesis of diseases and the mechanisms of action for therapies can be elucidated by identifying biomarkers, analyzing the metabolic pathway, discovering drug-target interactions, and so on. Recently, metabolic profiling has attracted great interest for biomarker discovery and for assessing the holistic effects of many therapeutics used in TCM [11–16].

Network pharmacology, a system biology-based methodology, is a new approach to highlight a holistic thinking and systematical theory of interactions among drugs, targets, and diseases, which is also shared with TCM theory. The remarkable feature of network pharmacology is the “multicomponent therapeutics, network target” [17–19]. In the network pharmacology-based drug research, a biological network of a disease and a pharmacological network of the candidates are established. So, the network pharmacology applied to TCM research would provide a novel methodology and opportunity for screening bioactive ingredients, synergistic drug combinations [20], and biomarkers, revealing mechanisms of action and exploring scientific evidence of herbs [21] or herbal formulae on the basis of complex biological systems [22].

Here, for the first time, we studied the network targets and pathway of SFZYD bioactive ingredients. Furthermore, the plasma and urine metabolomics of cold coagulation blood stasis syndrome, which was induced by epinephrine and cold evil, and the therapeutic effects of SFZYD in rats were investigated. We used the ultra-high-performance liquid chromatography in tandem with a time-of-flight mass spectrometry (UHPLC-TOF/MS) based metabolomic approach to elucidate the metabolic profiles and phenotype changes between normal rats and model rats and to identify potential biomarkers. A multivariate statistical analysis was used to investigate the pathological variations of cold coagulation blood stasis syndrome and to explore the therapeutic effects of SFZYD.

## 2. Materials and Methods

### 2.1. Materials

Ten crude herbs, *Angelicae sinensis Radix*, *Chuanxiong Rhizoma*, *Paeoniae Radix Rubra*, *Cinnamomi Cortex*, *Foeniculi Fructus*, *Zingiberis Rhizoma*, *Myrrha, Trogopterpri Faeces*, *Typhae Pollen*, and *Corydalis Rhizoma*, were purchased from Minxian (Gansu), Pengzhou (Sichuan), Chifeng (Neimeng), Yulin (Guangxi), Wuwei (Gansu), Yulin (Guangxi), Guangdong, Changzhi (Shanxi), Jiangsu (Yixing), and Songyang (Zhejiang), respectively. The corresponding author authenticated all of the raw materials, and the herbal drugs were verified according to the Chinese pharmacopoeia (Chinese pharmacopoeia, 2010). The voucher specimens (no. NJUTCM-20110818-20110827) were deposited in the Jiangsu Key Laboratory for TCM Formulae Research of Nanjing University of Chinese Medicine. 

 HPLC grade acetonitrile was purchased from Tedia (Fairfield, OH, USA), and AR grade formic acid was purchased from the Shanghai Reagent Company (Shanghai, China). Ultrapure water for UHPLC analysis was prepared using a Millipore water purification system (Millipore, Milford, MA, USA) and filtered with 0.22 *μ*m membranes prior to use. Sodium citrate (no. 20071107) and epinephrine hydrochloride (no. 0808231) were obtained from Tianjin Biochem Pharmaceutical CO., LTD., and Tianjin King York Amino Acid CO., LTD., respectively.

### 2.2. Preparation of SFZYD Extract

*Angelicae sinensis Radix*, *Chuanxiong Rhizoma*, *Paeoniae Radix Rubra*, *Cinnamomi Cortex*, *Foeniculi Fructus*, *Zingiberis Rhizoma*, Myrrha,* Trogopterpri Faeces*, *Typhae Pollen*, and *Corydalis Rhizoma* were mixed at a weight ratio of 3 : 1 : 2 : 1 : 0.5 : 2 : 1 : 3 : 1 : 1, respectively. The mixture (1.5 kg) was decocted with 15 L of water for 2 h. The filtrates were collected, and the residues were decocted in 12 L of water for 1.5 h. The filtrates from each decoction were combined and concentrated to 1.5 L at 70°C. The concentrated solution was dried with a vacuum, and 28.9 g extract of Shaofu Zhuyu decoction (SFZYD) was produced.

### 2.3. Network Targets and Pathway Prediction of SFZYD Bioactive Ingredients

**Figure 1:**
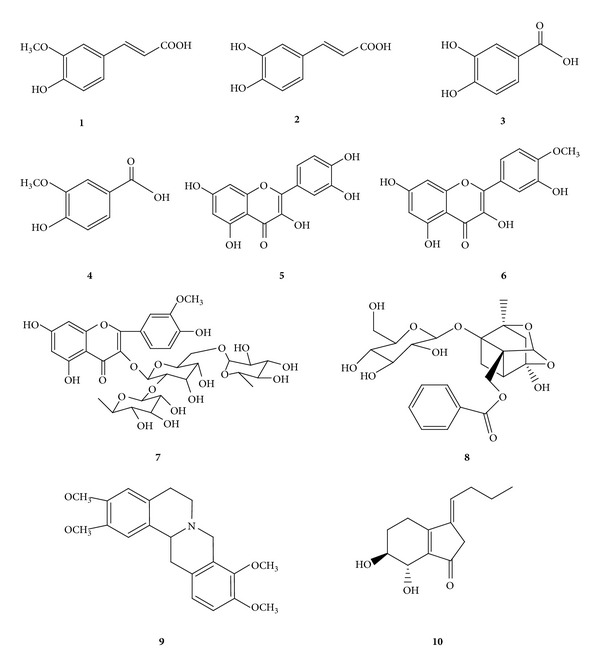
The chemical structures of the ten target compounds ((**1**) ferulic acid; (**2**) caffeic acid; (**3**) protocatechuic acid; (**4**) vanillic acid; (**5**) quercetin; (**6**) isorhamnetin; (**7**) typhaneoside; (**8**) paeoniflorin; (**9**) tetrahydropalmatine; (**10**) senkyunolide I).

Based on our previous studies [23, 24], the ten compounds absorbed into blood were selected to predict the biological targets. The mol2 files of ten compounds are imported into PharmMapper database (http://59.78.96.61/pharmmapper/) to predict the targets, then the target numbers were entered into the database (http://www.uniprot.org/, http://bioinfo.capitalbio.com/mas3/),  and the Kyoto Encyclopedia of Genes and Genomes (KEGG) database (http://www.genome.jp/kegg/) was used to annotate and analyze the pathway. The chemical structures of ten compounds from SFZYD were shown in Figure 1.

### 2.4. Animal Handling Procedure and Drug Treatment

Female Sprague-Dawley (SD) rats (180 ± 10 g) were purchased from Nanjing University of Chinese Medicine (rodent license no. SCXK 20080033). The rats were housed under standard laboratory conditions, and food and tap water were provided ad libitum. Experimental procedures were carried out in accordance with the Guide for the Care and Use of Laboratory Animals, and before the animal experiments were carried out, the procedures were approved by the Laboratory Animal Center of Nanjing University of Chinese Medicine.

The experimental groups (*n* = 6) were as follows: (1) normal control (NC), (2) cold- and epinephrine-induced CCBS syndrome (CCBS), and (3) CCBS model rats with SFZYD treatment. The model rats were put into ice water (0°C~1°C) for 5 min daily for 7 days. On the 8th day, they received two subcutaneous injections of hypodermic epinephrine (1 mg·kg^−1^) at 4-hour intervals. Simultaneously, model rats were administered SFZYD (10.08 g kg^−1^; 10 times the clinical dose for humans) via gastric irrigation once daily for 7 days. The dose of SFZYD was chosen based on the clinical application dosage of 45 g/day/60 kg body weight. The rats in the normal control group were treated with an equal volume of distilled water as a vehicle control.

### 2.5. Samples Collection and Preparation

Blood samples were collected in heparinized tubes on the 8th day after the injection of epinephrine. They were then anticoagulated in natrium citricum, centrifuged at 15000 ×g for 10 min, and stored at −20°C until analysis. Urine samples were collected in 12-hour intervals every day for 10 days, then centrifuged at 15000  ×g for 10 min and stored at −20°C until analysis.

Two hundred microliters of plasma was added to 600 *μ*L of acetonitrile, and this mixture was vortexed for 30 s and centrifuged at 15,000 ×g for 10 min to obtain the supernatant. Prior to analysis, the urine samples were thawed at room temperature and centrifuged at 15000 ×g for 10 min. The supernatant liquid (1 mL) was added to 3 mL of acetonitrile, vortex mixed for 30 s, and centrifuged at 15000 ×g  for 10 min to obtain the supernatant. The samples were processed and analyzed in a random order.

The plasma and urine supernatants were removed and evaporated to dryness in a 40°C water bath under a gentle stream of nitrogen. The residues were reconstituted in 200 *μ*L mobile phase of 70% acetonitrile-water solution, centrifuged at 15000 ×g for 5 min, and filtered through a 0.22 *μ*m membrane filter. The filtrates were transferred to an autosampler vial and stored at 4°C. A 5 *μ*L aliquot of each plasma or urine sample was injected for LC/MS analysis. The samples were analyzed in a random order.

### 2.6. Model Assessment

The hemorheology indexes, coagulation function, and metabolic profiling changes were calculated to evaluate the success of the CCBS syndrome model in rats. The hemorheology indexes of whole blood viscosity and plasma viscosity were measured according to a previously described method [25]. The coagulation function index, including thrombin time (TT), prothrombin time (PT), activated partial thromboplastin time (APTT), and fibrinogen (FIB), was determined to assess the CCBS syndrome model. Thrombin time (TT) was determined using a coagulometer (Model LG-PABER-I, Steellex Co., China). Shortly after adding the thrombin solution, the coagulometer was started and TT was recorded. To establish the standard curve of TT and thrombin concentration, TT was determined by incubating 40 *μ*L of plasma for 3 min at 37°C, and this was followed by the addition of 40 *μ*L of a thrombin solution (different concentrations in Tris-HCl butter, pH 7.4) and 20 *μ*L of solvent for 3 min at 37°C. TT was examined using the previously described method and converted into thrombin concentration using the indicated regression equation. Prothrombin time (PT), activated partial thromboplastin time (APTT), and fibrinogen content (FIB) were examined with commercial kits, following the manufacturer's instructions with slight modification. PT was determined by incubating 40 *μ*L of plasma solution for 3 min at 37°C, followed by the addition of 40 *μ*L of thromboplastin agent and 20 *μ*L of sample. APTT was determined by incubating 10 *μ*L of the sample solution and 50 *μ*L of plasma with 50 *μ*L of APTT-activating agents for 3 min at 37°C, followed by the addition of 50 *μ*L of CaCl_2_. FIB was determined by incubating 10 *μ*L of plasma with 90 *μ*L of imidazole buffer for 3 min at 37°C, followed by the addition of 50 *μ*L of FIB agent and 10 *μ*L of the sample solution. 5-HT, NA, and *β*-EP were determined according to the methods of ELISA kits described.

### 2.7. UPLC-QTOF/MS and UPLC-QqQ/MS Analysis

Chromatography was performed on an AcQuity UHPLC system (Waters Corp., Milford, MA, USA) with a conditioned autosampler at 4°C. The separation was carried out on an AcQuity UHPLC BEH C_18_ column (50 mm × 2.1 mm i.d., 1.7 *μ*m; Waters Corp., Milford, MA, USA), which was maintained at 35°C. The mobile phase consisted of 0.1% formic acid (HOOCH) in water as solvent A and acetonitrile (ACN) as solvent B. The gradient conditions of the mobile phase were as follows: 0~4 min, A: 98%~85%; 4~9 min, A: 85%~70%; 9~12 min, A: 70%~65%; 12~15 min, A: 65%; 15~18 min, A: 65%~50%; 18~21 min, 50%~25%; 21~22 min, 25%~20%; 22~26 min, 20%~5%; 26~28 min, 5%; and 29~31 min, 98%. The flow rate was 0.40 mL min^−1^, and the sample injection volume was 2 *μ*L.

Mass spectrometric detection was carried out on an AcQuity Synapt mass spectrometer equipped with an electrospray ionization (ESI) interface (Waters, Milford, MA, USA). The ESI source was operated in negative and positive ionization modes. The ionization source conditions were as follows: capillary voltage of 3.0 kV, source temperature of 120°C, and desolvation temperature of 350°C. The sampling cone voltage was set to 30 V, the extraction cone voltage was 2.0 V (for plasma sample) or 0.7 V (for urine sample), the trap collision energy was 6.0 V, the transfer collision energy was 4.0 V, the trap gas flow was 1.50 mL min^−1^, and the ion energy was 1.0 V. Nitrogen and argon were used as cone and collision gases, respectively. The cone and desolvation gas flow were 50 and 600 L h^−1^, respectively. The scan time was 0.5 s (for plasma sample) or 0.2 s (for urine sample), and an interval scan time of 0.02 s was used throughout, with a collision energy of 6 eV. The mass spectrometric data were collected from *m/z* 100 to 1000 in centroid mode. Leucine-enkephalin was used as the lock mass, generating an [M+H]^+^ ion (*m/z* 556.2771) and an [M−H]^−^ ion (*m/z* 554.2615) at a concentration of 200 pg mL^−1^ and a flow rate of 100 *μ*L min^−1^. Dynamic range enhancement was applied throughout the MS experiment to ensure that accurate mass measurements were obtained over a wider dynamic range.

### 2.8. Metabolomic Data Processing and Multivariate Analysis

UPLC/MS data were detected and noise-reduced in both the UPLC and MS domains such that only true analytical peaks were selected for further processing by the software. A list of the peak intensities detected was then generated for the first chromatogram using the Rt-*m/z* data pairs as identifiers. The resulting normalized peak intensities form a single matrix, with Rt-*m/z *pairs for each file in the dataset. All processed data from each chromatogram were normalized and Pareto-scaled prior to the multivariate statistical analysis.

All data from the plasma and urine samples were processed using the MarkerLynx application manager for MassLynx 4.1 and MarkerLynx software (Waters Corp., Milford, USA). The intensity of each ion was normalized with respect to the total ion count to generate a data matrix consisting of the retention time, *m/z* value, and normalized peak area. The multivariate data matrix was analyzed using *EZ* info software (Waters Corp., Milford, USA). Unsupervised segregation was examined with a principal component analysis (PCA) using Pareto-scaled data. A partial least squared discriminant analysis (PLS-DA) and an orthogonal partial least-squared discriminant analysis (OPLS-DA) were used to identify the various metabolites responsible for the separation between the model and normal groups. Potential biomarkers of interest were extracted from the S-plots that were constructed following the OPLS-DA analysis, and the biomarkers were chosen based on their contribution to the variation and correlation within the dataset.

 An internal 5-fold cross-validation was carried out to estimate the performance of the PLS-DA models. The calculated *R* ^2^ *Y*_(cum)_ estimates how well the model represents the fraction of explained Y-variation, and *Q*_(cum)_^2^ estimates the predictive ability of the model. Models are considered excellent when the cumulative values of *R* ^2^ *Y* and *Q* ^2^ are greater than 0.8. In addition to cross-validation, 200 permutation tests were also performed to validate the model. The variable importance in the projection (VIP) value is a weighted sum of squares of the PLS weights that reflects the relative contribution of each *X* variable to the model. The variables with VIP > 1 were considered to be influential for sample separation in the score plots generated from PLS-DA analysis [26]. Ultimately, different metabolic features associated with the model group and the SFZYD treatment group were obtained based on cutoff points of both VIP values from a 5-fold cross-validated PLS-DA model and critical *P values* from a univariate analysis. In addition, the corresponding fold change was calculated to show the degree of variation in metabolite levels between groups.

### 2.9. Biomarker Identification and Construction of the Metabolic Pathway

The identities of the potential biomarkers were confirmed by comparing their mass spectra and chromatographic retention times with the available reference standards and a full spectral library containing MS/MS data obtained in the positive and/or negative ion modes. The Mass Fragment application manager (Waters MassLynx v4.1, Waters Corp., Milford, USA) was used to facilitate the MS/MS fragment ion analysis through the use of chemically intelligent peak-matching algorithms. This information was then used to search multiple databases, either in-house or using online data sources, including ChemSpider database (http://www.chemspider.com), Mass Bank (http://www.massbank.jp/), PubChem (http://ncbi.nlm.nih.gov/), and MetFrag (http://msbi.ipb-halle.de/MetFrag/).

 To identify the affected metabolic pathways, a construction, interaction, and pathway analysis of potential biomarkers was performed using MetPA (http://metpa.metabolomics.ca./MetPA/faces/Home.jsp) based on several database sources, including the KEGG (http://www.genome.jp/kegg/), Human Metabolome Database (http://www.hmdb.ca/), SMPD (http://www.smpdb.ca/), and METLIN (http://metlin.scripps.edu/). Potential biological roles were evaluated by an enrichment analysis using MetaboAnalyst.

### 2.10. Statistical Analysis

All quantitative data analyses were performed using the SPSS 11.5 software package for Windows. Significance was determined using one-way analyses of variance (ANOVAs), followed by Student's *t*-test. The results were expressed as the mean ±SD. *P *values less than 0.05 were considered significant.

## 3. Results and Discussion

### 3.1. Potential Targets and Pathway Analysis

**Figure 2:**
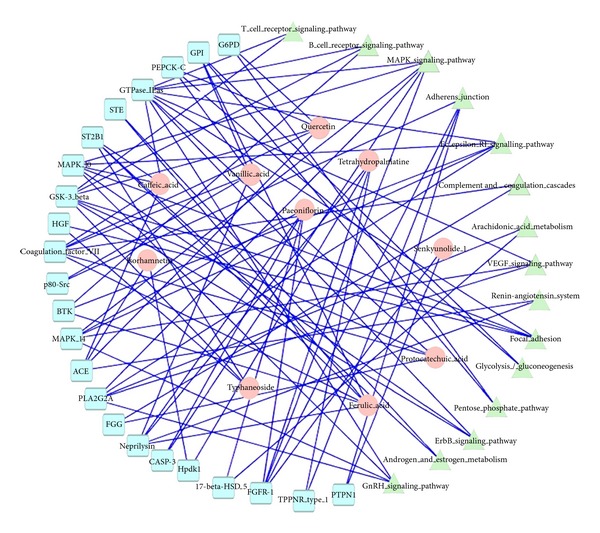
The relationships between the ingredients, targets, and pathways based on network biology.

The 10 compounds that confirmed the absorption into the blood were imported into the database of PharmMapper to analyze the reverse docking. The results showed that 23 important potential protein targets were found, and these targets were put into the KEGG pathway annotation, the 15 pathways were discovered. Among these pathways there were 8 pathways related to inflammation and immunological stress. They are arachidonic acid metabolism, MAPK, adherens junction, focal adhesion, Fc epsilon RI, VEGF, B cell receptor signaling pathway, and T-cell receptor signaling pathway, respectively. The pathways associated with hormone metabolism are including androgen and estrogen metabolism, GnRH signaling pathway, and ErbB signaling pathway.  Figure 2 showed the relationships between the ingredients, targets, and pathways.

### 3.2. Changes in Blood Indexes and Model Evaluation

Blood viscosity is the measure of how thin or thick the blood fluid is, and it reflects the blood flow and blood flow resistance. When blood is thick, blood flow is sluggish and there is an increased resistance, which tends to hinder normal energy metabolism and can lead to functional disorders in organs and tissues. In this experiment, whole blood viscosity and plasma viscosity indexes were determined for CCBS model rats. The whole blood viscosity (at high and low shear rates of 200 s^−1^, 30 s^−1^, 5 s^−1^, and 1 s^−1^) and plasma viscosity in model rat plasma were significantly increased (*P* < 0.05 or 0.01) (Table 1), which suggests that the blood of these CCBS syndrome rats is in a viscous or stasis state. SFZYD significantly regulated whole blood viscosity at low shear rates (5 s^−1^ and 1 s^−1^) in model rats (*P* < 0.05) at 5.04 and 10.08 g kg^−1^ doses, respectively. At a dose of 10.08 g kg^−1^, SFZYD also significantly decreased whole blood viscosity at high shear rates (30 s^−1^ and 200 s^−1^) and plasma viscosity (*P* < 0.05 or *P* < 0.01).

Coagulation is one important index for evaluating the state of blood stasis syndrome. The coagulation pathway comprises the complex interaction of many elements of the endothelium, coagulation factors, and platelets. Among these, thrombin plays a pivotal role in blood stasis syndrome. Thrombin acts as a multifunctional serine protease that is generated in response to vascular injury, and it catalyzes the proteolytic cleavage of the soluble plasma-protein fibrinogen to form insoluble fibrin, leading to clot formation. Thrombin also serves as a potent platelet agonist and amplifies its own generation via feedback activation of several steps in the coagulation cascade.

The PT and APTT assays were developed based on theories and the specific need for testing, without complete knowledge of all the proteins involved in coagulation. Our data showed that in model rats, the TT, PT, APTT, and FIB were remarkably decreased compared with those of normal rats. Furthermore, SFZYD prolonged TT and PT significantly (*P* < 0.05) in the model group (Table 2). At a dosage of 10.08 g kg^−1^, SFZYD also showed activity toward APTT.

The determined data of 5-HT, NA, and *β*-EP were listed in Table 3. The results showed that the contents of 5-HT and NA in both brain tissue and plasma and of *β*-EP in plasma significantly increased in model rat (*P* < 0.05), while the content of *β*-EP in brain tissue was decreased in model rat (*P* < 0.05). After SFZYD treatment, the abnormal state was regulated remarkably (*P* < 0.05 or *P* < 0.01).

 Moreover, we evaluated the model by analyzing metabolic profiling changes based on urine metabolomic data. The base peak intensity (BPI) chromatograms of the urine samples collected in positive ion mode during the eight days of animal model preparation are shown in (see Figure S1 Supplementary Material available online at http://dx.doi.org/10.1155/2013/901943). The results revealed that during the first three days of animal model preparation,there was no obvious departure of urine metabolic profiling, as determined by PCA, while pronounced changes were observed after 5 and 7 days of preparation, especially on the eighth day after the injection of hypodermic epinephrine, when there was a remarkable change in the metabolic profile (see Figure S2).

### 3.3. Metabolic Profiling Analysis

**Figure 3:**
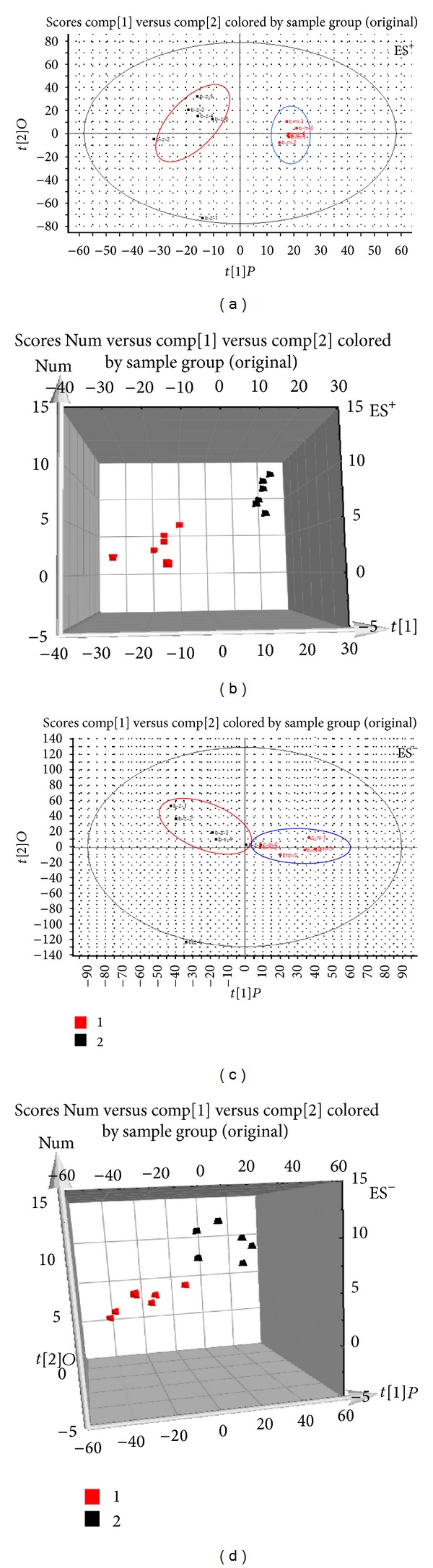
PLS-DA model results for samples obtained from the plasma of the normal and model groups and analyzed in positive and negative ion modes (ES^+^: (a) 2-D plot; (b) 3-D plot; ES^−^: (c) 2-D plot; (d) 3-D plot).

**Figure 4:**
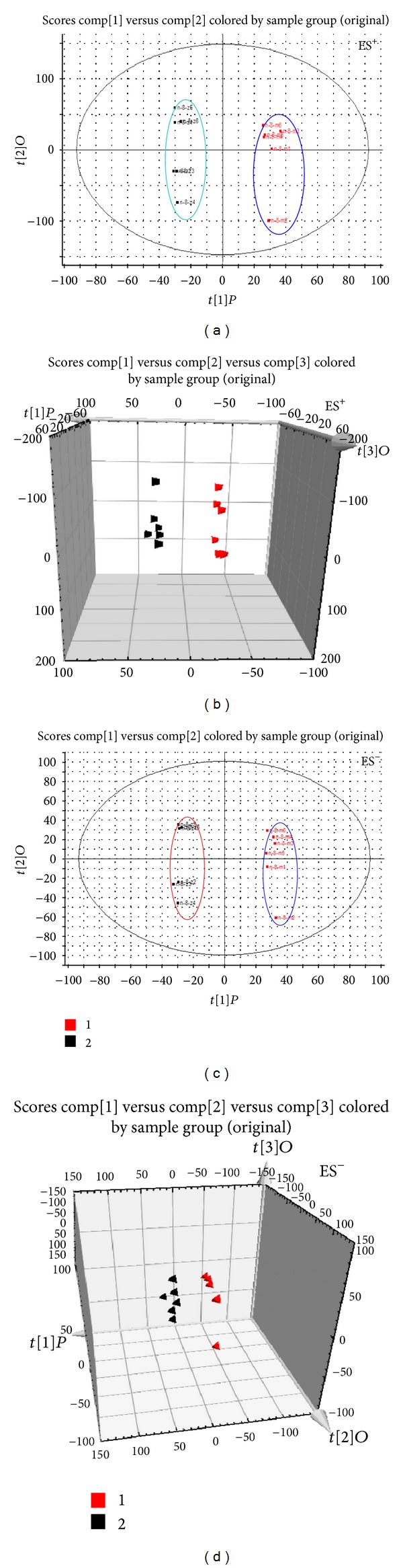
PLS-DA model results for the samples obtained from the urine of the normal and model groups and analyzed in positive and negative ion modes (ES^+^: (a) 2-D plot; (b) 3-D plot; ES^−^: (c) 2-D plot; (d) 3-D plot).

**Figure 5:**
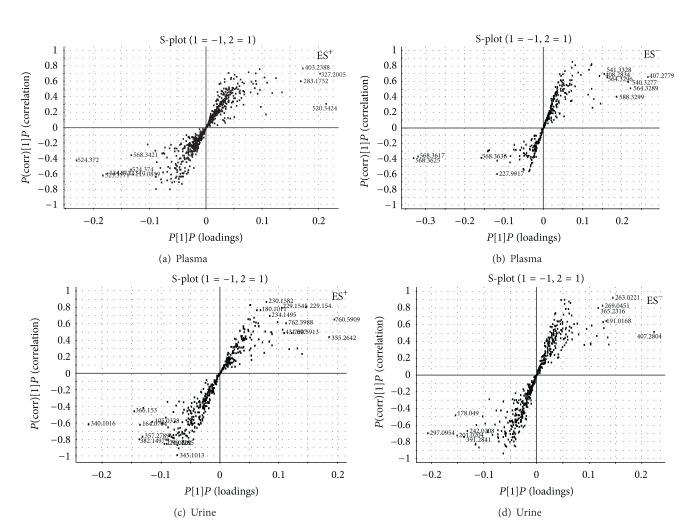
S-plot of the OPLS-DA model for the plasma and urine samples from the normal versus model groups (plasma: (a) ES^+^ mode; (b) ESI^−^ mode; urine: (c) ES^+^ mode; and (d) ES^−^ mode).

Typical base peak intensity (BPI) chromatograms in positive and negative ion modes of plasma and urine samples collected from normal and model rats are shown in Figure S3. The unsupervised PCA model was used to separate the plasma and urine samples into two blocks, respectively, between the normal group and the model group in positive and negative ion modes (Figures 3(a), 3(b), 3(c), 3(d), 4(a), 4(b), 4(c), and 4(d)). The supervised OPLS-DA, which could improve biomarker discovery and separate the samples into two blocks, was applied to obtain better discrimination between the two groups. The OPLS-DA score plot analysis of the chromatographic data identified the plasma and urine samples of the normal group and the model group based on the differences in their metabolic profiles, which suggested that the metabolic profiles were significantly changed as a result of CCBS syndrome (Figures 5(a), 5(b), 5(c), and 5(d)). The recognition of a different pattern indicates that the endogenous metabolites have changed in CCBS syndrome model rats.

OPLS-DA distinguished normal rat and model rat cohorts with 100% sensitivity and no less than 95% specificity using a leave-one-out algorithm. The *R* ^2^ *Y* of this PLS-DA model was 0.926 and 0.917 (plasma samples) and 0.947 and 0.922 (urine samples) in the positive and negative ion modes, respectively. The *Q* ^2^ of the model was 0.849 and 0.853 (plasma samples) and 0.823 and 0.837 (urine samples), respectively. These results indicate that the OPLS-DA models were reliable.

### 3.4. Metabolites Identification

The ions furthest away from the origin contribute significantly to the clustering of the two groups, and they may be regarded as the potential biomarkers in model rats. Q-TOF was used to determine the precise molecular mass of these compounds within measurement errors (<5 ppm). The potential elemental composition, degree of unsaturation, and fractional isotope abundance of the compounds were obtained. The presumed molecular mass was searched in the METLIN database, Chemspider, Human Metabolome Database, and other databases to identify the possible chemical constitutions, and MS/MS data were used to determine the possible structures of these ions.

 Potential markers were extracted from S-plots and constructed following the OPLS analysis. Then, markers were chosen on the basis of their contribution to the variation and correlation within the data set. In the plasma, ten endogenous metabolites that contributed to the separation of the model rats and the normal rats were identified by comparing their molecular ion information and the corresponding fragments of product ions with authentic standards (Table 4). To illustrate the identification of metabolites, we will describe the ion at *t*_*R*_ = 15.97 min (*m/z* 496.3450) as an example. This ion may contain an odd number of nitrogen atoms because its precise molecular weight is 496.3383, and its molecular formula was speculated to be C_24_H_50_NO_7_P, based on the analysis of its elemental composition and fractional isotope abundance. In the positive ion spectrum, the main fragment ions that were analyzed via MS/MS screening were observed at *m/z* 478.3290, 419.2279, 313.1615, 184.0786, 125.9252, and 104.1071, which could be the [M+H]^+^ due to the loss of –H_2_O, –C_3_H_10_NO, –C_5_H_13_NO_4_P, –C_19_H_37_NO_2_, –C_22_H_44_NO_3_, and –C_20_H_42_NO_4_P, respectively. Finally, to define its structure, we searched the HMDB database, and the metabolite was tentatively identified as lysophosphatidylcholine (16 : 0) [LPC (16 : 0)].

Compared with normal rats, four metabolites were upregulated (*P* < 0.05) in CCBS syndrome model rats, including 5-dehydro-4-deoxy-*D*-glucarate, 5*α*-tetrahydrocorticosterone, PC (13 : 0/0 : 0), and 17-phenyl trinor PGF_2*α*_ methyl ester. Alternatively, six metabolites were significantly downregulated (*P* < 0.05) in CCBS syndrome model rats, including LysoPC (22 : 5 (7Z,10Z,13Z,16Z,19Z)), LysoPC (17 : 0), PC (0 : 0/18 : 0), LysoPC (18 : 2(9Z,12Z)), LysoPC (16 : 0), and LysoPC (22 : 6 (4Z, 7Z, 10Z, 13Z, 16Z, 19Z)) (Table 4).

In urine samples, the significant variables that were identified in positive and negative ion modes are summarized in Table 5. Ten endogenous metabolites were tentatively identified using the methods described above. The metabolites of cholic acid, 3-methoxy-4-hydroxyphenylglycol sulfate, 5-dehydro-4-deoxy-*D*-glucarate, 5**α**-tetrahydrocortisol, and 13,14-dihydro PGF_2*α*_ were significantly upregulated (*P* < 0.05) in CCBS syndrome model rats, whereas the metabolites of 2-phenylethanol glucuronide, hippuric acid, 6-hydroxy-5-methoxyindole glucuronide, 2,8-dihydroxyquinoline-beta-D-glucuronide, and normeperidinic acid glucuronide were significantly downregulated (*P* < 0.05). The fact that different metabolites were altered in the plasma and urine may denote the potential of these metabolites as targeted biomarkers that can be used to differentiate between the CCBS syndrome and normal states.

### 3.5. Metabolic Pathway and Function Analysis

Metabolite profiling is the analysis of a group of metabolites that are related to a specific metabolic pathway in biological states. More detailed analyses of the most relevant pathways and networks for CCBS were performed using Metaboanalyst, which is a free, web-based tool that combines the results of powerful pathway enrichment analysis with the conditions of the study. Metaboanalyst and directed graph use the high-quality KEGG (http://www.genome.jp/kegg/) pathway database as the backend knowledgebase. Consequently, the identification of potential targets using a metabolic pathway analysis (impact value⩾0.10) with Metaboanalyst revealed that metabolites that were identified together are important for the host response to CCBS, and they are responsible for pentose and glucuronate interconversions and glycerophospholipid metabolism (see Figure S4). Distinct metabolic pathway analyses (impact value⩾0.10) were performed to identify pathways related to CCBS.

### 3.6. Therapeutic Effects of SFZYD

**Figure 6:**
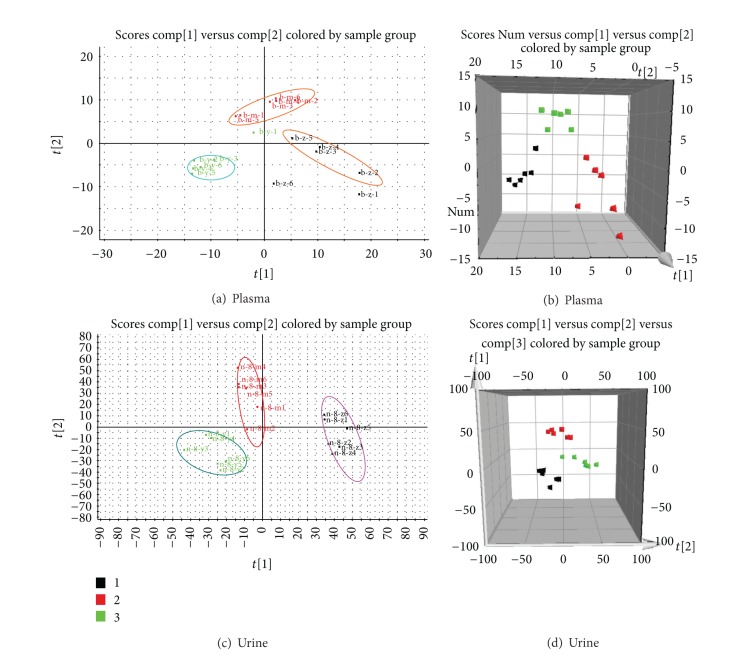
PLS-DA model results and loadings plots for the plasma and urine samples obtained from the normal, model, and treatment groups and analyzed in positive ion mode (Plasma: (a) 2-D plot and (b) 3-D plot; Urine: (c) 2-D plot and (d) 3-D plot).

**Figure 7:**
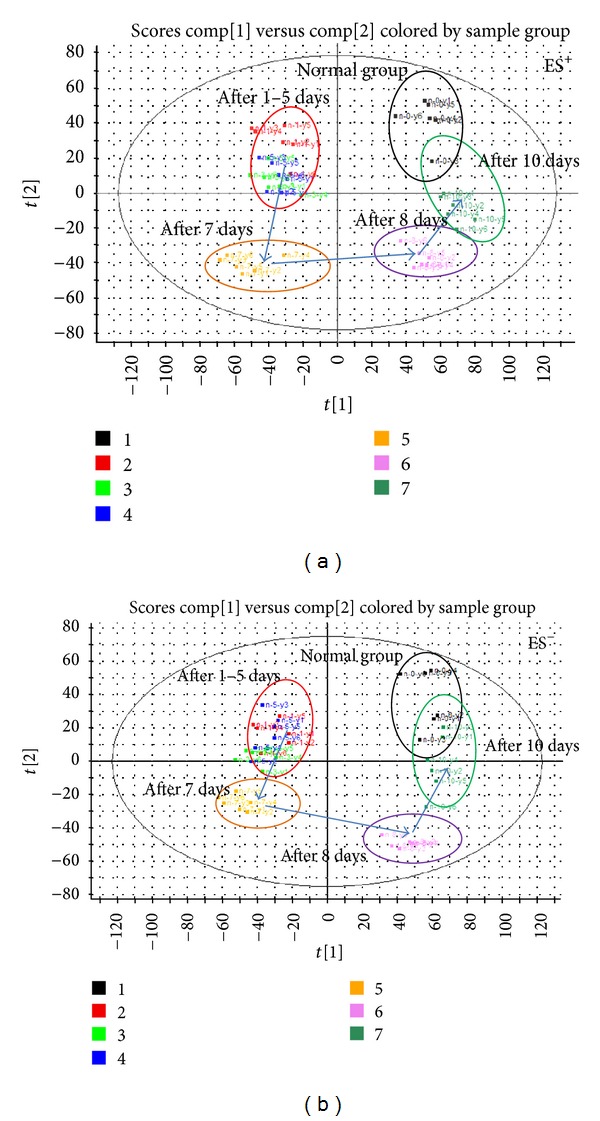
Urine metabolic profile changes after days 0, 1, 3, 5, 7, 8, and 10 of SFZYD treatment, as analyzed in positive and negative ion modes ((a) ES^+^ mode; (b) ES^−^ mode) [23].

**Figure 8:**
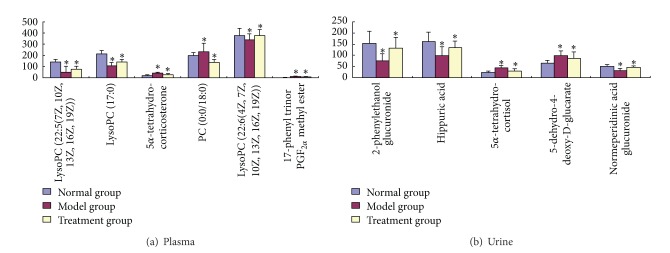
Changes in the relative quantities of targeted metabolites in the plasma and urine, identified in different groups. A two-tailed, parametric *t*-test was used to determine the significance of the changes for each metabolite, in relative quantities. Bars represent the mean relative metabolite concentration and standard deviations. *P < 0.05.

To more clearly characterize the effects of SFZYD on CCBS model rats, a PCA analysis was carried out to determine the changes before and after SFZYD treatment. The results revealed variations between the plasma and urine metabolic profiles of the model group, normal group, and SFZYD group (Figures 6(a), 6(b), 6(c), and 6(d)). To better understand the time-dependent effect of SFZYD, a PCA model was constructed to analyze all the data acquired from the normal group, predose group and treatment group at days 1, 3, 5, 7, and 10 in both positive and negative ion modes (Figures 7(a) and 7(b)). The spot observed in the treatment group at days 1–5 is close to that of the model group, indicating that the cold coagulation and blood stasis syndrome state is dominant. The spots observed in the treatment group at day 7 clustered near the center of the plot with a shift back toward the normal group, which might be an indication of the accumulated effect of SFZYD. The spot observed in the treatment group at days 8 and 10 ultimately approached the normal state, suggesting that SFZYD treatment had a positive therapeutic effect on the rats. Furthermore, the relative concentrations of 6 endogenous metabolites in the plasma and 5 endogenous metabolites in the urine were significantly affected by SFZYD (*P* < 0.05). All of these metabolites returned to normal levels after SFZYD treatment (Figures 8(a) and 8(b)). Thus, the efficient regulation of these potential biomarkers may account for the effects of SFZYD in model rats.

The prediction and identification of molecular markers (targets) and metabolic pathways have the potential to improve the diagnosis, prognosis, and therapy for ZHENG or diseases [27–30]. We utilized reverse docking method to predict the biology targets and pathway annotation. Furthermore a metabolomic approach to analyze the changes in the plasma and urine samples of CCBS syndrome model rats, normal rats, and SFZYD-treated rats was applied. We identified 20 endogenous metabolites (10 in the plasma and 10 in the urine) that were upregulated or downregulated (*P* < 0.05  or  0.01) in CCBS syndrome, including LysoPCs and glucuronide metabolites. Furthermore, 11 potential biomarkers (6 metabolites in the plasma and 5 metabolites the in urine) were regulated by SFZYD.

Studies using targeted metabolite analyses have already shown that alterations in critical CCBS syndrome metabolic pathways, such as glycerophospholipid metabolism (impact value 0.24) and pentose and glucuronate interconversions (impact value 0.27), are strongly associated with the development of CCBS syndrome. Phospholipid metabolism and glycometabolism were disturbed in the plasma and urine of CCBS rats, respectively. In urine, the levels of the metabolites of 2-phenylethanol glucuronide, hippuric acid, and normeperidinic acid glucuronide, which are all related to glycometabolism, were significantly decreased in model rats. The low level of glucuronide metabolites implies that energy metabolism decreased and the energy metabolism pathway increased, resulting in abnormal pentose and glucuronate interconversions. Previous studies have also reported that cold exposure increased energy expenditure by activating specific sympathetic pathways [31]. These results agreed well with the SFZYD bioactive ingredients prediction of network targets related to inflammation and immunological stress, hormone metabolism, glycometabolism, and coagulation cascade system.

Ephedrine is known to raise blood pressure, heart rate, and energy expenditure and to increase the levels of multiple circulating metabolites, including glucose, insulin, and thyroid hormones [31]. In this paper, cold and ephedrine mutually induced CCBS syndrome in model rats, and the level of phosphatidylcholine in the plasma decreased. Among the potential markers, 5**α**-tetrahydrocorticosterone and 17-phenyl trinor PGF2**α** methyl ester levels increased in the plasma of the model rats. 5**α**-Tetrahydrocorticosterone is a corticosteroid hormone, and the hypothalamic-pituitary-adrenal axis was activated in the CCBS model rats induced with cold and epinephrine. The excretion of corticotropin-releasing factor (CRF) by the hypothalamus and pituitarium leads to the release of adrenocorticotropic hormone, which acts on the adrenal cortex to promote the secretion of corticosteroid hormones [32]. Therefore, the combination of cold and ephedrine could change the metabolism of hormones in CCBS syndrome model rats. Moreover, the levels of LysoPC metabolites were decreased in the plasma of model rats.

It was reported that primary dysmenorrhea patients with CCBS syndrome have high levels of prostaglandin growth factor 2 (PGF2) in their menstrual fluid [33], and PGF2 stimulates myometrial contractions and ischemia and sensitizes nerve endings. In this paper, CCBS syndrome model rats had high levels of 17-phenyl trinor PGF_2*α*_ methyl ester, and phospholipid metabolism was disturbed, likely due to the inflammatory response. Future research will focus on the discovery of additional biomarkers using metabolomics platforms and the validation of explorative biomarkers.

In addition, 11 specific metabolites regulated by SFZYD were identified, including 5**α**-tetrahydrocortisol, 5-dehydro-4-deoxy-D-glucarate, 2-phenylethanol glucuronide, hippuric acid, and normeperidinic acid glucuronide in the urine and 5**α**-tetrahydrocorticosterone, PC (0 : 0/18 : 0), 17-phenyl trinor PGF_2*α*_ methyl ester, LysoPC (22 : 6(4Z, 7Z, 10Z, 13Z, 16Z, 19Z)), LysoPC (17 : 0), and LysoPC (22 : 6 (4Z, 7Z, 10Z, 13Z, 16Z, 19Z)) in the plasma. These biomarkers suggest that the pathogenesis of CCBS syndrome is closely related to glycometabolism and phospholipid metabolism. Based on these findings, further studies would be performed to validate the predicted targets and the changed metabolites and to elucidate the mechanisms underlying these alterations.

Our results also showed that SFZYD improved the status of hemorheology and blood viscosity, and it regulated the coagulation function of TT and APTT. These data imply that the state of CCBS syndrome is closely associated with blood coagulation function. This research also verified that ephedrine-induced platelet aggregation, gluconeogenesis, ischemia, and Ca^2+^ influx in vascular endothelial cells are mediated by CNGA2 channels [34–37]. So, the systems biology and network targets prediction are one of the most important trends for the development of traditional Chinese medicine [38–40].

## 4. Conclusions

In summary, the reverse docking method and metabolomics-based study provide a powerful approach to evaluate the effects of Chinese herbs and discover potential biomarkers (targets) via the prediction of biological targets and analysis of global changes in an individual's metabolic profile. Here, for the first time, we performed a comprehensive analysis of the network targets, pathways induced by SFZYD bioactive ingredients, and metabolic patterns of CCBS syndrome. Our findings suggest that the proposed approach would be helpful for establishing a suitable model to reasonably evaluate CCBS syndrome, explore its pathological mechanisms, and clarify the mechanisms of action of SFZYD.

## Supplementary Material

Figure S1: Figure S1 displays the TIC chromatography of the first, third, fifth, seventh, eighth day during animal model preparation.
Figure S2: Figure S2 showed the metabolic profiling changes during the eight days of animal model preparation.
Figure S3: Figure S3 displays the typical base peak intensity (BPI) chromatograms in positive and negative ion modes of plasma and urine samples collected from normal and model rats.
Figure S4: Figure S4 showed the summary of pathway analysis with MetPA in plasma and urine.

## Figures and Tables

**Table 1:** Changes in the whole blood and plasma shear viscosity of model rats and SFZYD-treated rats ($n=6,\;\overset{-}{x}\pm s$).

	Whole blood viscosity (*η*b/mPa·s)				plasma viscosity (*η*b/mPa·s)
	200 (s^−1^)	30 (s^−1^)	5 (s^−1^)	1 (s^−1^)	
Normal group	4.01 ± 0.13	4.95 ± 0.17	7.58 ± 0.46	14.85 ± 1.54	2.45 ± 0.16
Model group	4.24 ± 0.10^Δ^	5.46 ± 0.19^ΔΔ^	8.99 ± 0.59^ΔΔ^	19.19 ± 2.01^ΔΔ^	3.08 ± 0.60^Δ^
SFZYD group (5.4 g kg^−1^)	4.15 ± 0.18	5.20 ± 0.23	8.19 ± 0.49*	16.61 ± 1.48**	2.84 ± 0.32
SFZYD group (10.8 g kg^−1^)	4.00 ± 0.11*	5.10 ± 0.18*	7.89 ± 0.53**	15.86 ± 1.63**	2.67 ± 0.21*

^Δ^ *P* < 0.05, ^ΔΔ^ *P* < 0.01 normal group versus model group; *P < 0.05 model group versus SFZYD group.

**Table 2:** Changes in the plasma coagulation function of model rats and SFZYD-treated rats ($n=6\;,\overset{-}{x}\pm s$).

	TT (s)	PT (s)	APTT (s)	FIB (g/L)
Normal group	31.5 ± 3.9	20.0 ± 2.3	47.2 ± 4.7	26.9 ± 1.8
Model group	22.7 ± 2.0^ΔΔ^	14.4 ± 1.7^Δ^	34.8 ± 3.1^Δ^	18.4 ± 2.9^ΔΔ^
SFZYD group (5.4 g kg^−1^)	28.2 ± 2.3*	19.9 ± 1.9*	36.6 ± 3.6	20.9 ± 2.3
SFZYD group (10.8 g kg^−1^)	30.9 ± 3.3**	20.9 ± 1.2**	38.9 ± 2.8*	21.4 ± 2.5

^Δ^ *P* < 0.05, ^ΔΔ^ *P* < 0.01 normal group versus model group; *P < 0.05 model group versus SFZYD group.

**Table 3:** The contents of 5-HT, NA, and *β*-EP in plasma and brain tissue of model rats and treatment group ($n=6,\;\overset{-}{x}\pm s$).

	5-HT (ng/mL)		NA (ng/mL)		*β*-EP (ng/mL)	
	Brain tissue	Plasma	Brain tissue	Plasma	Brain tissue	Plasma
Normal group	144.39 ± 30.36	467.71 ± 86.01	3.92 ± 0.72	15.23 ± 2.66	0.28 ± 0.10	2.57 ± 0.45
Model group	259.38 ± 74.48^#^	549.9 ± 90.1^#^	4.8 ± 1.3^#^	18.5 ± 1.9^#^	0.15 ± 0.08^#^	3.47 ± 1.68^#^
SFZYD group (5.4 g kg^−1^)	183.49 ± 89.99*	492.79 ± 72.14*	4.33 ± 1.46	16.95 ± 5.11*	0.24 ± 0.09*	2.38 ± 1.42*
SFZYD group (10.8 g kg^−1^)	153.56 ± 76.32**	458.24 ± 81.56**	3.85 ± 1.45*	15.87 ± 4.36**	0.34 ± 0.12*	2.56 ± 1.89*

5-HT: 5-hydroxytryptamine; NA: Noradrenaline; *β*-EP: *β*-endorphin. Data were expressed as Mean ± SEM. Means between the normal group, model group, low-dose SFZYD-treated group, and high-dose SFZYD-treated group. Significant differences when compared with the model group *P < 0.05, **P < 0.01 and compared with the normal group ^#^ *P* < 0.05.

**Table 4:** Identification of differentially expressed metabolites in the plasma that may account for the discrimination between normal and model rats.

No.	Metabolites	Formula	Obsd. [M + H]^+^/[M − H]^−^ (*m*/*z*)	Content variance^c^	FC^d^	*P* ^ e^ value
1	LysoPC (22:5 (7Z, 10Z, 13Z, 16Z, 19Z))	C_30_H_52_NO_7_P	568.3436^a^	↓	−2.33	0.018
2	5-Dehydro-4-deoxy-D-glucarate	C_6_H_8_O_7_	191.0197^a^	↑	2.56	0.022
3	LysoPC (17:0)	C_25_H_52_NO_7_P	508.3403^a^	↓	−1.42	0.030
4	5*α*-Tetrahydrocorticosterone	C_21_H_34_O_4_	349.2378^a^	↑	2.56	0.035
5	PC (13:0/0:0)	C_21_H_44_NO_7_P	452.2774^a^	↑	1.25	0.021
6	17-phenyl trinor PGF2*α* methyl ester	C_24_H_34_O_5_	404.2438^b^	↑	2.01	0.017
7	PC (0:0/18:0)	C_26_H_54_NO_7_P	524.3720^b^	↓	−1.35	0.042
8	LysoPC (18:2 (9Z, 12Z))	C_26_H_50_NO_7_P	520.3424^b^	↓	−1.56	0.030
9	LysoPC (16:0)	C_24_H_50_NO_7_P	496.3450^b^	↓	−1.78	0.016
10	LysoPC (22:6 (4Z, 7Z, 10Z, 13Z, 16Z, 19Z))	C_30_H_50_NO_7_P	568.3421^b^	↓	−2.60	0.026

^a^Observed at ES^−^ mode [M − H]^−^; ^b^observed at ES^+^ mode [M + H]^+^ *.*

^c^↑: content increased; ↓: content decreased.

^d^Fold change was calculated as the ratio of the mean metabolite levels between two groups. A positive value of fold change indicates a relatively higher concentration of metabolites, while a negative value of fold change indicates a relatively lower concentration of metabolites in model rats as compared to normal rats.

^e^ *P* values were calculated from two-tailed Mann-Whitney *U* test with a threshold of 0.05.

**Table 5:** Identification of differentially expressed metabolites in the urine that may account for the discrimination between normal and model rats.

No.	Metabolites	Formula	Obsd. [M + H]^+^/[M − H]^−^ (*m*/*z*)	Content variance^c^	FC^d^	*P* ^ e^ value
1	Cholic acid	C_24_H_40_O_5_	407.2804^a^	↑	1.56	0.035
2	2-Phenylethanol glucuronide	C_14_H_18_O_7_	297.0954^a^	↓	−1.69	0.026
3	Hippuric acid	C_9_H_9_NO_3_	178.0491^a^	↓	−1.81	0.040
4	3-Methoxy-4-hydroxyphenylglycol sulfate	C_9_H_12_O_7_S	263.0221^c^	↑	2.53	0.025
5	5-Dehydro-4-deoxy-D-glucarate	C_6_H_8_O_7_	191.0168^a^	↑	2.40	0.016
6	5*α*-Tetrahydrocortisol	C_21_H_34_O_5_	365.2316^a^	↑	3.70	0.023
7	6-Hydroxy-5-methoxyindole glucuronide	C_15_H_17_NO_8_	340.1016^b^	↓	−3.79	0.012
8	2,8-Dihydroxyquinoline-beta-D-glucuronide	C_15_H_15_NO_8_	338.0847^b^	↓	−2.42	0.028
9	Normeperidinic acid glucuronide	C_18_H_23_NO_8_	382.1497^b^	↓	−3.21	0.037
10	13,14-dihydro PGF2*α*	C_20_H_36_O_5_	357.2724^b^	↑	2.01	0.028

^a^Observed at ES^−^ mode [M − H]^−^; ^b^observed at ES^+^ mode [M + H]^+^ *.*

^c^↑: content increased; ↓: content decreased.

^d^Fold change was calculated as the ratio of the mean metabolite levels between two groups. A positive value of fold change indicates a relatively higher concentration of metabolites while a negative value of fold change indicates a relatively lower concentration of metabolites in model rats as compared to normal rats.

^e^ *P* values were calculated from two-tailed Mann-Whitney *U* test with a threshold of 0.05.

## Abbreviations

PD: Primary dysmenorrhea
SFZYD: Shaofu Zhuyu decoction
MS/MS: Tandem mass spectrometry
OPLS: Orthogonal partial least squares
PCA: Principal components analysis
PLS-DA: Partial least-squares discriminant analysis
RT: Retention time
TOFMS: Time-of-flight mass spectrometry
UPLC: Ultraperformance liquid chromatography.
