# Supplementary material for: Network-Based Biomarkers for Cold Coagulation Blood Stasis Syndrome and the Therapeutic Effects of Shaofu Zhuyu Decoction in Rats
Source: Evid Based Complement Alternat Med. 2013 Oct 21;2013:901943. doi: 10.1155/2013/901943 (PMC3818846; doi:10.1155/2013/901943)
Supplement: Supplementary file 1 — Figure S1: Figure S1 displays the TIC chromatography of the first, third, fifth, seventh, eighth day during animal model preparation. Figure S2: Figure S2 showed the metabolic profiling changes during the eight days of animal model preparation. Figure S3: Figure S3 displays the typical base peak intensity (BPI) chromatograms in positive and negative ion modes of plasma and urine samples collected from normal and model rats. Figure S4: Figure S4 showed the summary of pathway analysis with MetPA in plasma and urine. [file 901943.f1.pdf]

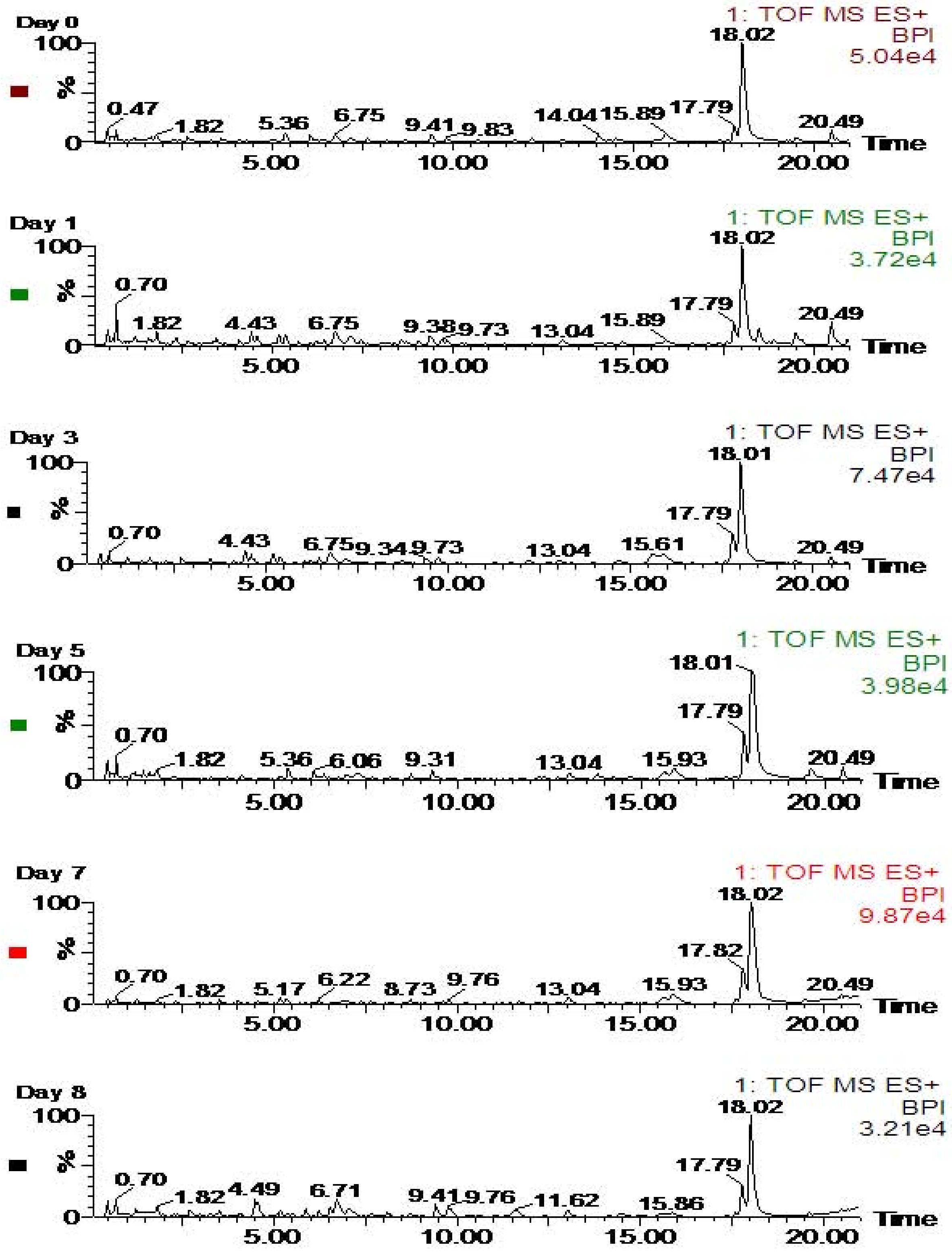
Day 0
1: TOF MS ES+
BPI
5.04e4
18.02
0.47
1.82
5.36
6.75
9.41
9.83
14.04
15.89
17.79
20.49
Day 1
1: TOF MS ES+
BPI
3.72e4
18.02
0.70
1.82
4.43
6.75
9.38
9.73
13.04
15.89
17.79
20.49
Day 3
1: TOF MS ES+
BPI
7.47e4
18.01
0.70
4.43
6.75
9.34
9.73
13.04
15.61
17.79
20.49
Day 5
1: TOF MS ES+
BPI
3.98e4
18.01
17.79
0.70
1.82
5.36
6.06
9.31
13.04
15.93
20.49
Day 7
1: TOF MS ES+
BPI
9.87e4
18.02
17.82
0.70
1.82
5.17
6.22
8.73
9.76
13.04
15.93
20.49
Day 8
1: TOF MS ES+
BPI
3.21e4
18.02
17.79
0.70
1.82
4.49
6.71
9.41
9.76
11.62
15.86
100
%
0
5.00
10.00
15.00
20.00
Time

Fig. S1

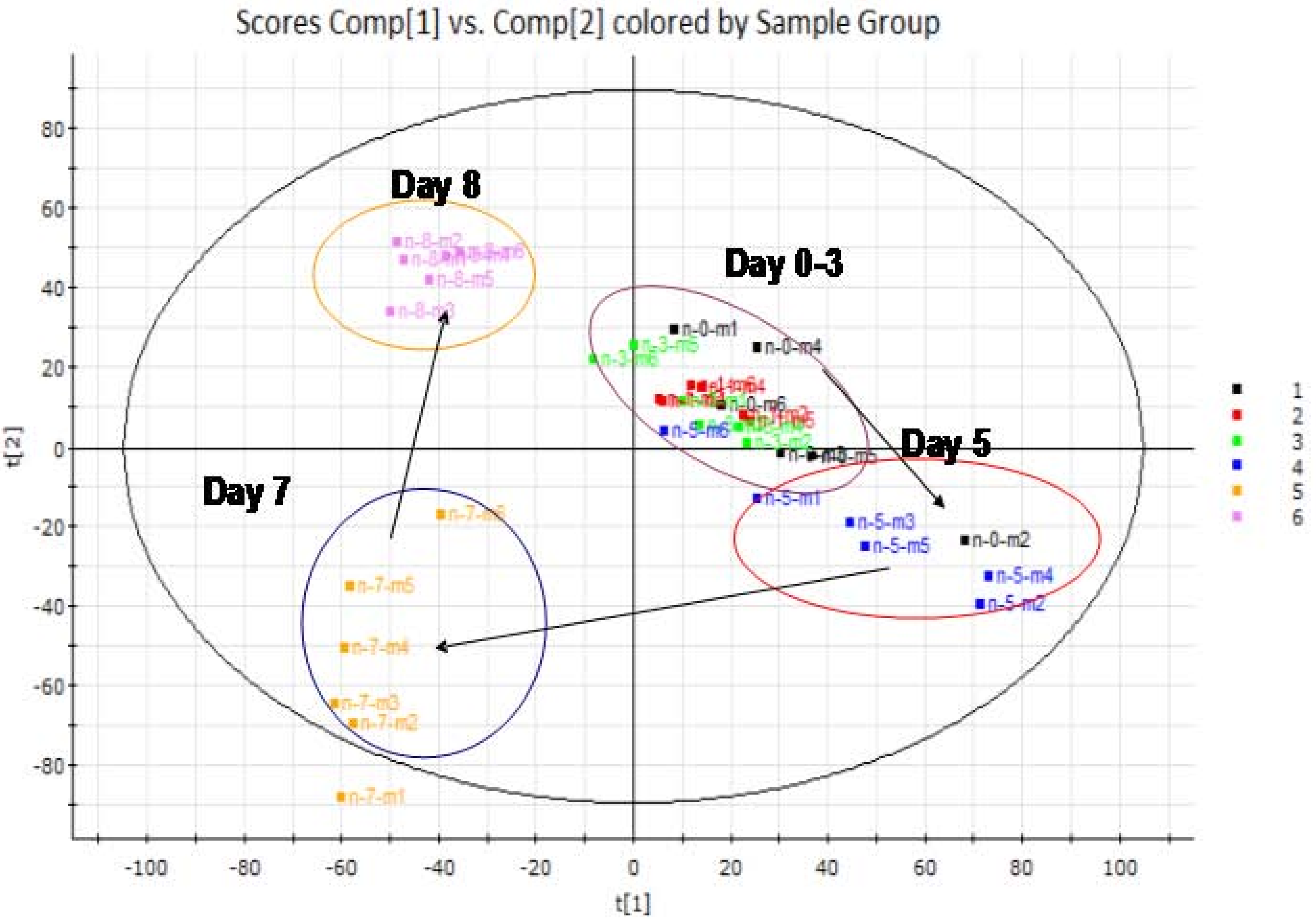

Scores Comp[1] vs. Comp[2] colored by Sample Group
Day 8
Day 0-3
Day 5
Day 7
t[1]
t[2]
1
2
3
4
5
6

Fig. S2

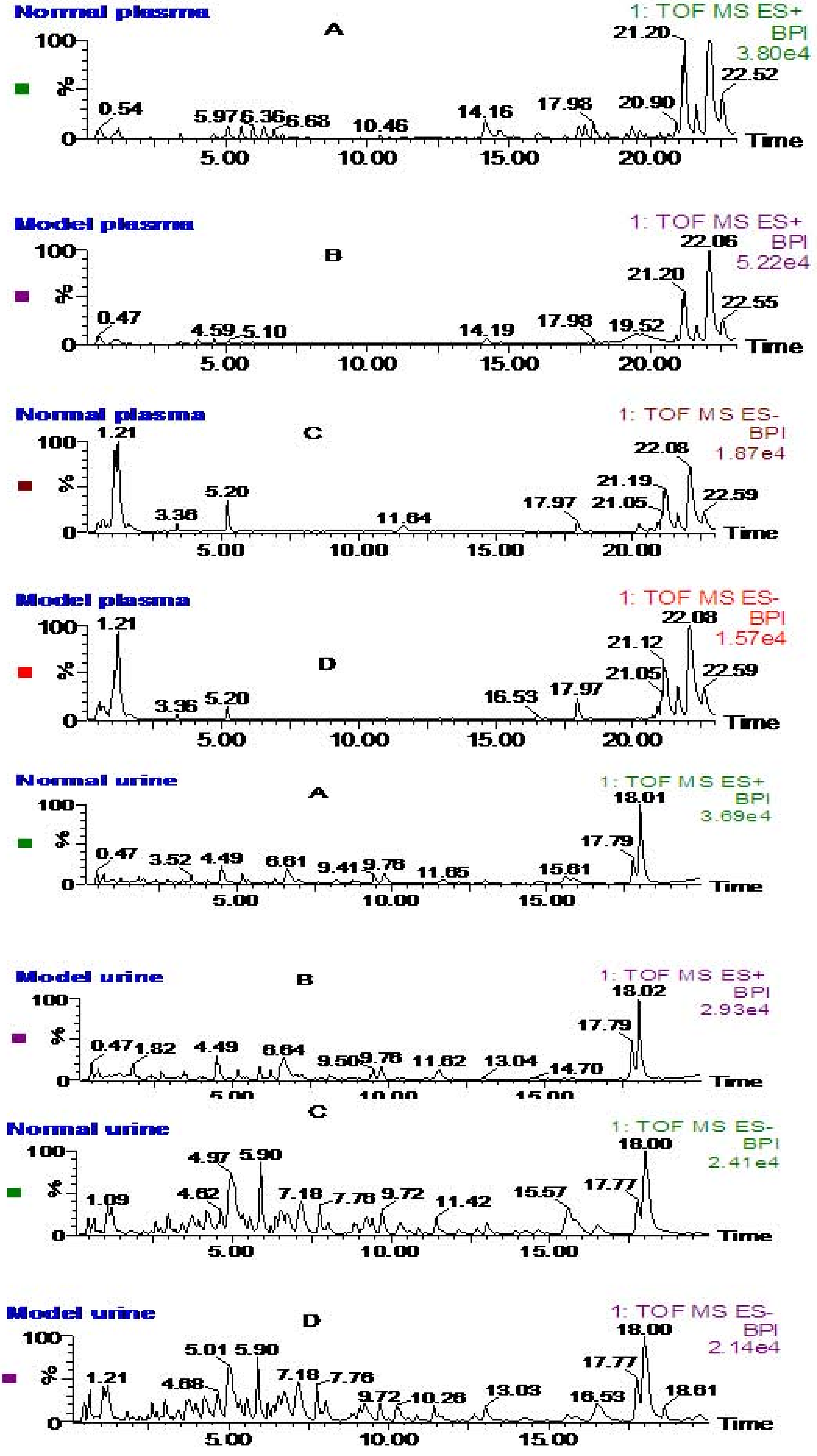
Normal plasma
A
1: TOF MS ES+
BPI
3.80e4
21.20
22.52
0.54
5.97
6.36
6.68
10.46
14.16
17.98
20.90
Time
5.00
10.00
15.00
20.00
Model plasma
B
1: TOF MS ES+
BPI
5.22e4
22.06
21.20
22.55
0.47
4.59
5.10
14.19
17.98
19.52
Normal plasma
C
1: TOF MS ES-
BPI
1.87e4
1.21
3.36
5.20
11.64
17.97
21.05
21.19
22.08
22.59
Model plasma
D
1: TOF MS ES-
BPI
1.57e4
1.21
3.36
5.20
16.53
17.97
21.05
21.12
22.08
22.59
Normal urine
A
1: TOF MS ES+
BPI
3.69e4
18.01
17.79
0.47
3.52
4.49
6.61
9.41
9.76
11.65
15.61
Model urine
B
1: TOF MS ES+
BPI
2.93e4
18.02
17.79
0.47
1.82
4.49
6.64
9.50
9.76
11.62
13.04
14.70
Normal urine
C
1: TOF MS ES-
BPI
2.41e4
18.00
17.77
1.09
4.62
4.97
5.90
7.18
7.76
9.72
11.42
15.57
Model urine
D
1: TOF MS ES-
BPI
2.14e4
18.00
17.77
1.21
4.68
5.01
5.90
7.18
7.76
9.72
10.26
13.03
16.53
18.61
100
%
0

Fig. S3

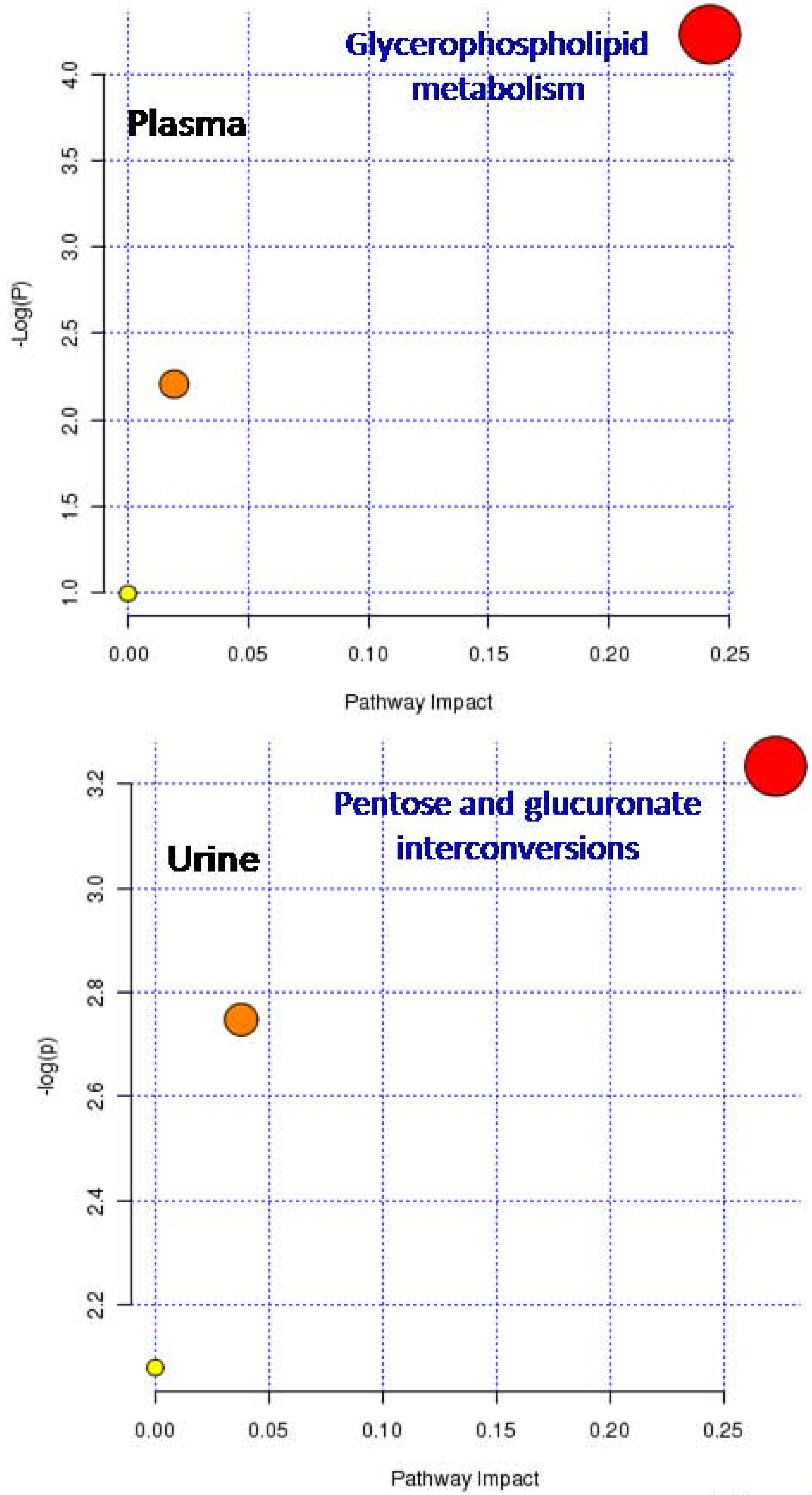
Glycerophospholipid
metabolism
Plasma
-Log(P)
4.0
3.5
3.0
2.5
2.0
1.5
1.0
0.00
0.05
0.10
0.15
0.20
0.25
Pathway Impact
Pentose and glucuronate
interconversions
Urine
-log(p)
3.2
3.0
2.8
2.6
2.4
2.2
0.00
0.05
0.10
0.15
0.20
0.25
Pathway Impact

Fig. S4
